# Our Public Institutions.—The State Insane Asylum

**Published:** 1881-06

**Authors:** 


### Editorial.

### OUR PUBLIC INSTITUTIONS.—THE STATE INSANE ASYLUM.

The ripple of excitement, originating in another part of the State, in regard to the management of Asylums for the Insane, and finally reaching our city, has passed away. The secular Press, in its attempt to array the profession and the public against the great humanitarian work carried on in these institutions, has failed to accomplish its purpose. The legislative committees delay their reports, presumably for the reason that there is "no cause of action." The medical profession, individually and collectively, may now, with an appearance at least of disinterestedness and impartiality, bear their testimony as to the necessity of these agencies in the special work of treating mental, and their concomitant diseases, and also as to the management of institutions, devoted to this specialty. With this object, and, also, with a view to gain information in regard to our public institutions, we lately visited the Buffalo State Asylum for the Insane and availed ourselves of the opportunity extended by its accomplished medical officers to carefully inspect every part of this costly and extensive structure.

We frankly acknowledge that our visit impressed one fact upon our minds, that the Empire State has a just reason to be proud of its public institutions, many of which—and in this foremost class we place our State Insane Asylum—are commensurate in their appointments, plans, organization and the costliness of their structures, with the wealth and extent of the great commonwealth they represent. This asylum has cne special advantage over any like institution on the continent in the facilities offered for the classification of the insane. It is constructed in five distinct and separate buildings, in addition to the central and administration building, connected only by fire-proof corridors, making each of the six magnificent and massive structures separate and almost isolated. The buildings are arranged with the most ample conveniences for the healthfulness and comfort ot the patients, the single rooms, and also the dormitories are well lighted, thoroughly ventilated, rather expensively furnished, the wards large and roomy, and presenting an appearance of adaptation to the requirements of this special class of diseases.

Modern pathology, in its development of the relations of mental to physical disorders has transformed the Insane Asylum from a prison for individual restraint and for the protection of communities, to a hospital for the scientific treatment of patients. The physical basis of insanity is an established principle in medical science, and the indications of treatment have become comparatively plain and well defined. Acting upon the principle that a sound mind can exist only in a sound body, the treatment of insanity is at length founded on a rational and scientific basis; hence, physical exercise is here prescribed, the dietary is regulated, the habits of the individual watched and corrected, so that the body, being invigorated under appropriate hygienic laws, affords, with its returning strength a healthier instrument for mental operations. Accuracy in diagnosis is under this theory, as capable of attainment as in other departments of medicine. We were gratified and, indeed, instructed with the scientific and rational views, held by the Superintendent, Dr. Andrews, for whose familiarity with this subject in its manifold phases, we are most happy to bear our united testimony.

Our inquiries were extended to the sources of revenue for the support of the Asylum, and we learned, that it is derived from two sources: 1st., public; 2d, private. The public revenue, if we may be permitted to use this term, is derived from county authorities, which are held responsible for the maintenance of patients, who are without means for their own support. This class always constitutes a large proportion of cases in charge of asylums, devoted to the treatment of insanity. The second class includes patients, who have the pecuniary ability to defray all expenses attending their care and treatment. For this class, the Asylum requires the payment of $6.00 per week, and upwards, according to the financial ability of the individual. The State provides the necessary buildings and all appurtenances required for this great and important work, while it will be seen the current expenses are met by county authorities and by individuals.

Such is a very hasty sketch of the visit we were privileged to make to this important institution, with the more prominent features of its administrative and professional management. We find abundant evidence to render an emphatic verdict of approval and endorsement of the able management of the Superintendent and his assistants, and commend the institution to the profession of the state. His reputation, prior to assuming the responsible position he now holds, is an ample guaranty of the position he will seek to attain in the management of the trust now reposed in him. We are also gratified to witness the increasing proficiency and popularity of our young friend, Dr. Wm. D. Granger, the first assistant physician, whose appointment was warmly endorsed by this Journal.

The State Asylum for the Insane, located in this city, is one of the necessities, which the growth in population and wealth of this section of the state, has long demanded. It is now established and doing its work with great efficiency and success. It claims the support of the profession. It yields advantages to a large class of patients, that, heretofore, were enjoyed only at great inconvenience and expense. It invites the profession and the public to an examination of its facilities, and offers through the high professional attainments of its medical staff, and the careful treatment given to those, who seek its protection as a means of cure, advantages, which, we think are unsurpassed in the successful management of all forms of mental disorders.

